# Co_3_O_4_ Nanoparticle-Modified Porous Carbons with High Microwave Absorption Performances

**DOI:** 10.3390/nano13061073

**Published:** 2023-03-16

**Authors:** Shuangyin Zeng, Shaojie Han, Xiaotian Sun, Li Wang, Yanfeng Gao, Zhang Chen, Haitao Feng

**Affiliations:** 1 School of Materials Science and Engineering, Shanghai University, Shanghai 200444, China; 2 Key Laboratory of Comprehensive and Highly Efficient Utilization of Salt Lake Resources, Qinghai Institute of Salt Lakes, Chinese Academy of Sciences, Xining 810008, China

**Keywords:** biomass porous carbon, Co_3_O_4_, microwave absorbing, impedance matching, dielectric loss

## Abstract

Carbon materials derived from natural biomaterials have received increasing attention because of their low cost, accessibility, and renewability. In this work, porous carbon (DPC) material prepared from D-fructose was used to make a DPC/Co_3_O_4_ composite microwave absorbing material. Their electromagnetic wave absorption properties were thoroughly investigated. The results show that the composition of Co_3_O_4_ nanoparticles with DPC had enhanced microwave absorption (−60 dB to −63.7 dB), reduced the frequency of the maximum reflection loss (RL) (16.9 GHz to 9.2 GHz), and had high reflection loss over a wide range of coating thicknesses (2.78–4.84 mm, highest reflection loss <−30 dB). This work provided a way for further research on the development of biomass-derived carbon as a sustainable, lightweight, high-performance microwave absorber for practical applications.

## 1. Introduction

Electronic technology is rapidly evolving these days, and the use of electromagnetic wave devices is becoming increasingly common, with extensive research and development in fields such as personal cell phones, radio communication devices, and radar [1,2]. These technologies provide great convenience in our lives while also causing significant harm in our daily lives [3,4]. As a result, various electromagnetic absorbing materials have been studied [5,6]; among them, carbon-based materials with excellent dielectric absorption and lightweight properties have been widely researched and developed [7,8,9,10].

High-performance carbon materials such as graphene and carbon nanotubes are limited for large-scale applications due to their complex and costly synthesis [11,12]. Biomass-derived porous carbon materials have rich pore structures, high specific surface areas, and adjustable pore sizes, in addition to their low cost, having attracted a lot of attention [13,14,15,16]. Our previous work used a KOH activation method to produce porous carbon (DPC) and developed a strong microwave absorbing porous carbon material [17]. It was found that the microporous structure in the DPC provides it with good impedance matching across a wide frequency range. The maximum RL achieved is 60.0 dB at 16.9 GHz (coating thickness is 2.30 mm), with an effective bandwidth as wide as 7.9 GHz. As a pure carbon material, DPC has shown excellent electromagnetic absorption properties. However, the frequency of its maximum RL is too high (16.9 GHz), and the maximum RL of it is sensitive to coat thickness.

Various other magnetic materials, such as Fe_3_O_4_, Co_3_O_4_, and NiFe_2_O_4_, have been introduced to improve/modulate the RL performance of porous carbon [18,19,20,21]. Among them, Co_3_O_4_ is a spinel-structured iron–oxygen compound with a black or gray–black powder that can be approximated as a compound formed by cobalt oxide (CoO) and cobaltic oxide (Co_2_O_3_) [22]. Ma et al. designed a three-dimensional graded Co_3_O_4_-modified graphene and used this multi-interface and porous flower-like structure to adjust the impedance matching to improve the absorbing performance with a maximum reflection loss of −61 dB at 11.2 GHz and an effective absorption bandwidth up to 4 GHz [23]. Liu et al. used thermal oxidation to convert cobalt-coated carbon fibers (Co/CFs) into cobalt oxide (Co_3_O_4_ and CoO)-coated carbon fiber (CoO_x_/CFs) composites with a maximum reflection loss of −45.16 dB at 13.41 GHz and an effective absorption bandwidth of 13.96 GHz [24]. Wen et al. homogeneously embedded carbon coated Co_3_O_4_ nanoparticles onto two-dimensional mesoporous graphitic carbon nanosheets and achieved good impedance matching via a significant synergistic effect, which facilitated microwave absorption [25]. Two-dimensional Co_3_O_4_@C@PGC nanosheets have a maximum reflection loss of −32.3 dB at a thickness of 2.3 mm and a microwave frequency of 11.4 GHz, demonstrating that Co_3_O_4_ can be well incorporated into the carbon materials and reduce the frequency of the maximum RL.

In this work, DPC/Co_3_O_4_ and a commercial coconut husk carbon (CHC)/Co_3_O_4_ were prepared by the hydrothermal introduction of Co_3_O_4_ particles. The microwave absorption performance of DPC600 and CHC before and after the addition of Co_3_O_4_ magnetic particles was evaluated, the parameters influencing the microwave absorption performance of the samples were identified, the influencing factors of the microwave absorption performance of the samples were investigated by varying the molar ratio of DPC/CHC to Co_3_O_4_ and the mass ratio of paraffin wax, and the optimal composite ratios of samples were further determined. This will broaden the pathway for further research on how to optimize the performance of microwave absorbing materials in the future.

## 2. Experimental Part

### 2.1. Materials

All chemicals were of analytical grade and used without further purification. D-fructose C_6_H_12_O_6_ and cobalt acetate (CH_3_CO_2_)_2_Co were purchased from Shanghai Aladdin Reagent Co., Ltd. (Shanghai, China) Coconut Shell Toner C was purchased from Jingchuan Environmental Protection Technology Co., Ltd. (Henan, China) Paraffin C_25_H_32_ was purchased from Beijing Bohui Innovation Biotechnology Co., Ltd. (Beijing, China) Potassium hydroxide KOH, hydrochloric acid (HCl) and anhydrous ethanol C_2_H_5_OH were purchased from Sinopharm Chemical Reagent Co., Ltd. (Shanghai, China)

### 2.2. Preparation of D-Fructose Porous Carbon

D-fructose porous carbon was prepared using the hydrothermal method as described in previous work [17]. That is, a 30 mL aqueous solution containing 3 g of D-fructose was put into a 40 mL stainless steel reactor and stirred on a constant temperature magnetic stirrer for 30 min. The reactor was kept at 220 °C for 24 h and then allowed to cool naturally. The prepared DC and KOH were mixed evenly according to the mass ratio of 1:4 and ground evenly in the mortar body, and the mixture was sent into the tube furnace under an N_2_ atmosphere at a heating rate of 3 °C/min to 600 °C for 2 h and then naturally cooled to room temperature, and the product was placed in dilute hydrochloric acid and deionized water, repeatedly cleaned to make the sample neutral, dried, and centrifuged to obtain a black powder, labeled as DPC600.

### 2.3. Preparation of D-Fructose Porous Carbon/Co_3_O_4_

**Figure 1:**
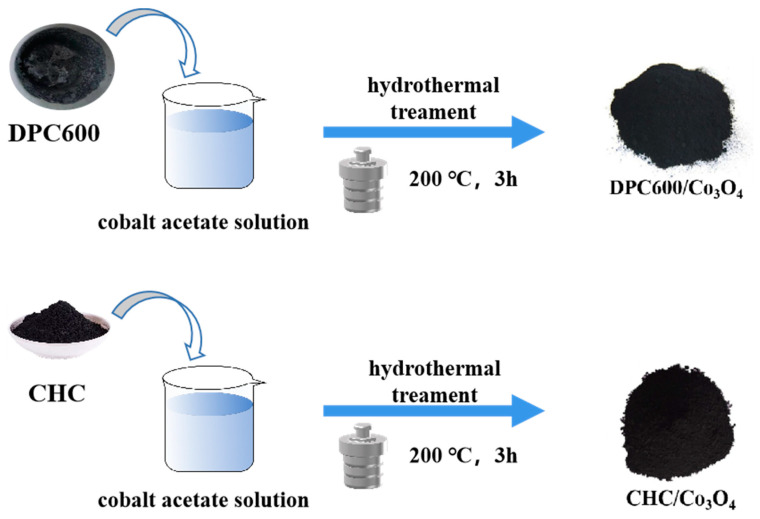
Schematic illustration of the synthesis of DPC/Co_3_O_4_ and CHC/Co_3_O_4_ samples.

A 30 mL aqueous solution containing 0.417 g (CH_3_CO_2_)_2_Co was put into a 40 mL PTFE reactor, and different quantities of DPC600 (0.0564 g, 0.084 g, and 0.1128 g) were added into it according to the molar ratio of Co_3_O_4_ to C (1:2, 1:3, and 1:4), respectively. The hydrothermal reaction was carried out after stirring for 30 min. The black precipitates were separated from the solvent by centrifugation at 8000 r/min after natural cooling at 200 °C for 3 h. Then, the black precipitate was washed with anhydrous ethanol and deionized water several times until the organic solvent was completely removed and was dried in a vacuum drying oven at 80 °C for 12 h. DPC600/Co_3_O_4_ complexes named DPC600/Co_3_O_4_-0.33, DPC600/Co_3_O_4_-0.25, and DPC600/Co_3_O_4_-0.20 were obtained. As a comparison, DPC600 was replaced with coconut shell toner (0.0564 g, 0.084 g, and 0.1128 g), and the subsequent steps were the same as before to obtain CHC/Co_3_O_4_-0.33, CHC/Co_3_O_4_-0.25, and CHC/Co_3_O_4_-0.20. The preparation process of composite materials is shown in Figure 1.

### 2.4. Characterization

The morphology of the samples was characterized using a field emission scanning electron microscope (SEM, a ZEISS Gemini 300 field emission SEM system). The crystallization properties were determined by a D8 Advance X-ray diffractometer (XRD) from Bruker, Germany, under Cu-Ka radiation. The specific surface area and pore structure of the porous materials were analyzed using a nitrogen isothermal adsorption–desorption analyzer (Micromeritics ASAP2460, Mack Instruments, USA). The complex permittivity and magnetic permeability were measured using a vector network analyzer (E5071C vector network analyzer from Agilent, USA) in the frequency range of 2–18 GHz. The samples to be tested and paraffin wax were mixed in mass ratios of 2:8, 3:7, and 4:6 to form coaxial rings with Φ_out_ of 7.0 mm and Φ_in_ of 3.04 mm and thicknesses in the range of 2–3 mm, and then the rings were placed in a vector network coaxial fixture for testing. All characterization and performance tests of the obtained samples were repeated at least three times to ensure reliability and reproducibility.

## 3. Results and Discussion

### 3.1. Characterization Results

**Figure 2:**
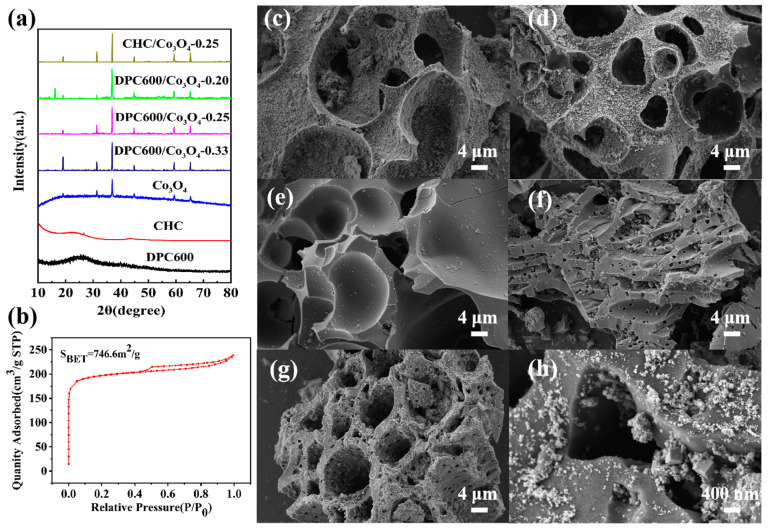
Physical characterization of the prepared samples. (**a**) XRD pattern of different samples: DPC600, CHC, and Co_3_O_4_ complexes with DPC600 molar ratios of 1:2 (DPC600/Co_3_O_4_-0.33), 1:3 (DPC600/Co_3_O_4_-0.25), and 1:4 (DPC600/Co_3_O_4_-0.20), and Co_3_O_4_ with coconut shell carbon powder molar ratio 1:3 (CHC/Co_3_O_4_-0.25); (**b**) nitrogen adsorption–desorption isotherms and the pore diameter distribution of CHC; SEM images of (**c**) DPC600/Co_3_O_4_-0.33, (**d**) DPC600/Co_3_O_4_-0.25, (**e**) DPC600/Co_3_O_4_-0.20, (**f**) CHC, (**g**) CHC/Co_3_O_4_-0.25, and (**h**) Co_3_O_4_ particles loaded on CHC.

The XRD patterns of the prepared samples are shown in Figure 2a. Both DPC600 and CHC had two distinct diffraction peaks, indicated they are amorphous carbon material; there was a strong diffraction peak on the (002) crystal plane around 2θ = 24° and a weak peak on the (100) crystal plane around 2θ = 42° [26,27]. XRD results of both DPC600/Co_3_O_4_ and CHC/Co_3_O_4_-0.25 indicated the existence of Co_3_O_4_ nanoparticles. The diffraction peaks at 19.0°, 31.2°, 36.8°, 44.8°, 59.3° 65.2°, and 78.4° corresponded to the (111), (220), (222), (400), (511), (440), and (533) crystal planes (JCPDS No. 43-1003), respectively; no others were detected. According to the width at half height of the XRD image of Co_3_O_4_, the particle size calculated by the Scheerer formula is in the order of tens of nanometers, which is consistent with the particles shown in the following SEM images.

DPC600 has an obvious three-dimensional interoperable porous skeletal carbon structure with the presence of a large number of pore structures as previous work. The specific surface area (S_BET_) of CHC was found to be 746.6 m^2^g^−1^, and S_micropores_ and S_external_ were 661.6 m^2^g^−1^ and 85.0 m^2^g^−1^, respectively, far from that of DPC600 (Figure 2b and Table 1) [17]. Moreover, DPC600 had more micropores (Smicropores, pore diameter 2 nm) than CHC; micropores contribute more to microwave absorption ability according to a prior study.

SEM images of the prepared samples are shown in Figure 2c–h. CHC shows an obvious and abundant pipeline structure, but the walls of the pipelines are obviously thicker than those of DPC (Figure 2f). DPC600/Co_3_O_4_ composites with varying Co_3_O_4_ loadings are depicted in Figure 2c–e. The white particles attached to the surface of the gray–black pore-like structure of DPC600 are Co_3_O_4_ crystals. As the content of Co_3_O_4_ rose, there were noticeably increased numbers of Co_3_O_4_ particles on the surface of porous carbon pores, and the Co_3_O_4_ particles gradually covered the whole surface area of DPC600. A dense coating formed when the content of Co_3_O_4_ rose to DPC600/Co_3_O_4_ = 2:1. The distribution state of Co_3_O_4_ particles in CHC/Co_3_O_4_ was similar to that in DPC600/Co_3_O_4_ composite samples (Figure 2g). Figure 2h shows Co_3_O_4_ particles loaded on CHC, and the size of Co_3_O_4_ particles was concentrated between tens of nanometers to hundreds of nanometers, the shape was cubic, and the small particle size was beneficial to the loading of Co_3_O_4_ into and around the pores of porous carbon to form an effective microwave absorption network.

### 3.2. Dielectric Properties of the Samples

Relative permittivity (*ε_r_* = *ε′* − *jε″*) and complex permeability (*μ_r_* = *μ′* − *jμ″*) are important parameters for microwave absorption properties. The electromagnetic parameters *ε′* and *μ′* represent the storage capacity of electrical and magnetic energy, respectively, while *ε″* and *μ″* refer to the loss capacity of electrical and magnetic energy, respectively [28,29]. The relative complex permittivity of samples was measured from a mixture of samples and paraffin with a mass ratio of 3:7 and a frequency range of 2–18 GHz [30].

**Figure 3:**
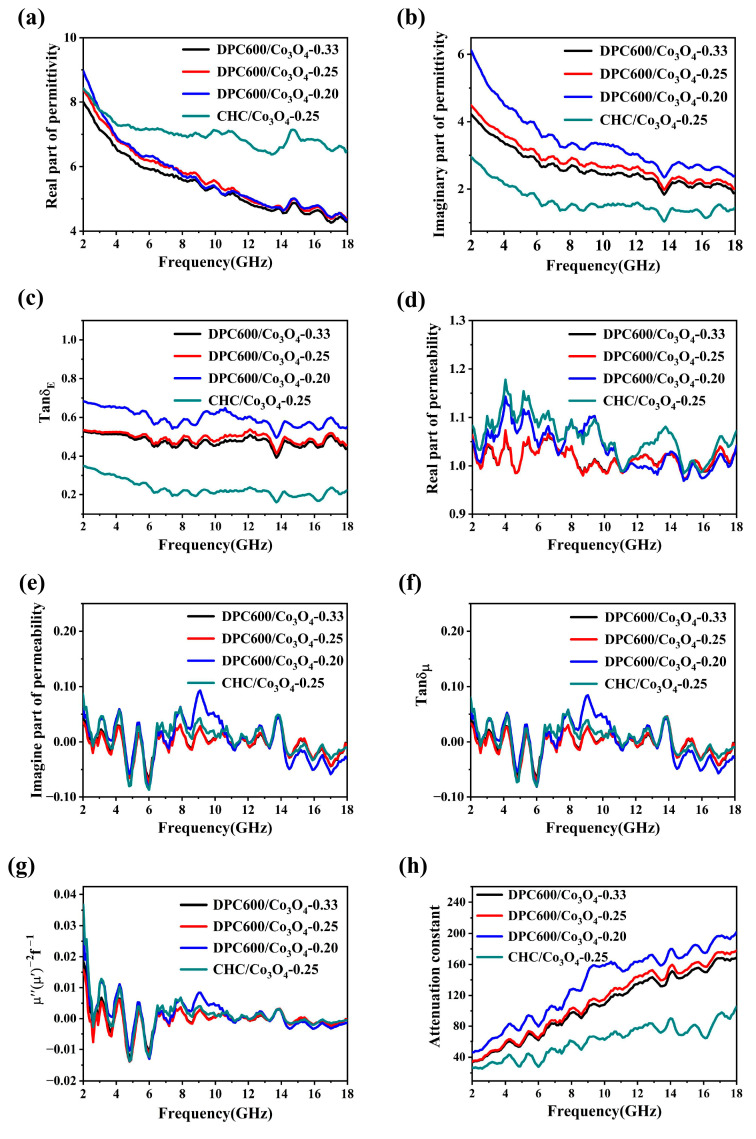
Characterization of the dielectric properties of prepared samples: (**a**) *ε′*, (**b**) *ε″*, (**c**) tan δ_E_, (**d**) *μ′*, (**e**) *μ″*, (**f**) tan δ*_μ_*, (**g**) *μ″*(*μ′*)^−2^*f*^−1^, and (**h**) attenuation content for DPC600/Co_3_O_4_-0.33, DPC600/Co_3_O_4_-0.25, DPC600/Co_3_O_4_-0.20, and CHC/Co_3_O_4_-0.25.

Figure 3a,b show a decreasing trend with increasing microwave frequency for composites with different molar ratios of DPC600 to Co_3_O_4_. The introduction of Co_3_O_4_ had a significant effect on the values of the imaginary parts of the dielectric constant of the material. With the gradual increase of the Co_3_O_4_ content, the imaginary parts of the dielectric constant in the frequency range of 2–18 GHz decreased. This was attributed to the poor conductivity and small carrier mobility of Co_3_O_4_. In the frequency range of 2–18 GHz, DPC/Co_3_O_4_-0.25 had smaller *ε′* but larger *ε″* than CHC/Co_3_O_4_-0.25. This was mainly due to the abundant pore structure of DPC600, which generated more dipole and interface polarization centers, thus enhancing the dielectric loss capacity.

Unlike the variations of *ε′* and *ε″*, the values of *μ′* and *μ″* for all four samples in Figure 3d,e were in a low range, with *μ′* fluctuating in the range of 1.0 to 1.2 and *μ″* varying from −0.10 to 0.10. In order to further explore the magnetic loss of the composite material after the addition of Co_3_O_4_, it is vital to explain the magnetic loss mechanism of DPC/Co_3_O_4_. The magnetic loss of composite materials to electromagnetic waves consists primarily of hysteresis loss, domain wall resonance, eddy current loss, natural resonance, and exchange resonance. Hysteresis loss and domain wall resonance occur in frequencies below 1 GHz and were not considered. When exploring the contribution of eddy current loss to magnetic loss, we can refer to the eddy current loss correlation coefficient *C*_0_, which can be calculated by Equation (1) [31] as follows:

(1) $$C_{0}=\mu^{\prime \prime }{\left(\mu^{\prime }\right)}^{-2}f^{-1}=\frac{2}{3}\pi \mu_{0}d^{2}\sigma$$

If the magnetic loss mode in the material is only eddy current loss, the value of the eddy current loss correlation coefficient *C*_0_ should be a constant. Figure 3g shows that *C*_0_ fluctuated with frequency in the 2–18 GHz frequency range. Thus, we believe that the magnetic loss generated by the injection of Co_3_O_4_ magnetic particles is attributable to its inherent resonance and exchange resonance, not only its eddy current loss. This conclusion is also consistent with the *μ″* multiple resonance peak in Figure 3e.

The reflection loss (RL) of the sample consists of two components: the dielectric loss in the absorber, and the impedance matching between the absorber and the free space [32]. The dielectric loss tangent (tan δ_E_ = *ε″/ε′*) is used to determine the strength of dielectric loss in absorbing materials [33]. A high dielectric loss factor necessitates both high *ε″* and low *ε′*. The dielectric loss angle of the composite varies between 0.5 and 0.7 at all concentrations, which is significantly greater than that of CHC/Co_3_O_4_-0.25, with CHC as the carbon matrix (Figure 3c). This also indicates that the dielectric loss capability of DPC600 is stronger than that of CHC.

The magnetic loss tangent (tan δ*_μ_* = *μ″/μ′*) is used to determine the strength of the magnetic loss of the absorbing materials [33]. All samples have small values of the complex permeability, which indicates that the samples have a weak magnetic loss capability. As can be seen in Figure 3f, the tan δ*_μ_* values of the samples are around 0. Furthermore, the values of tan δ_E_ for DPC/Co_3_O_4_ are higher than those of tan δ*_μ_*, suggesting dielectric loss comprised a major contribution to the electromagnetic wave attenuation over the frequency range from 2.0 to 18.0 GHz.

The electromagnetic wave attenuation ability of the material can also be evaluated by the attenuation constant (*α*); the larger *α* is, the stronger the material’s ability to lose electromagnetic waves. According to transmission line theory, the attenuation coefficient α can be calculated according to Equation (2) [34,35,36] as follows:

(2) α=2πf{(μ″ε″−μ′ε′)+[(μ″ε″−μ′ε′)2+(μ″ε″+μ′ε′)2]12}/c 

where *f* is the propagation frequency of electromagnetic waves, and *c* is the speed of light. It is obvious that higher values of *α* can be obtained with increasing values of *ε″* and *μ″*. It is clear from Figure 3h that the DPC600/Co_3_O_4_ composite had a higher *α*, which is consistent with the previously measured data, indicating that the DPC600/Co_3_O_4_ composite would have a strong electromagnetic attenuation capability. In addition, *α* became larger with the increase of carbon content in the composite, indicating the main contribution of dielectric loss to the attenuation of electromagnetic waves, which is consistent with the magnitude of the tangent of the dielectric loss angle and magnetic loss angle.

### 3.3. Electromagnetic Absorption Properties of the Samples

The EMA performance of the composites was evaluated by the reflection loss value (RL) determined from transmission line theory [37,38,39]. The absorption loss of the absorbing material for electromagnetic waves reached 90% when RL < −10 dB, and this frequency range is called the effective absorption band. The RL is calculated as follows (Equations (3) and (4)):

(3) $$Z_{\mathrm{in}}=Z_{0}\sqrt{\mu_{r}/\varepsilon_{r}}\tanh\left[\;j\left(2\pi fd/c\right)\sqrt{\mu_{r}\varepsilon_{r}}\;\right]$$

(4) $$\mathrm{RL}\left(\mathrm{dB}\right)=20\log\left|\left(Z_{\mathrm{in}}-Z_{0}\right)/\left(Z_{\mathrm{in}}+Z_{0}\right)\right|$$

**Figure 4:**
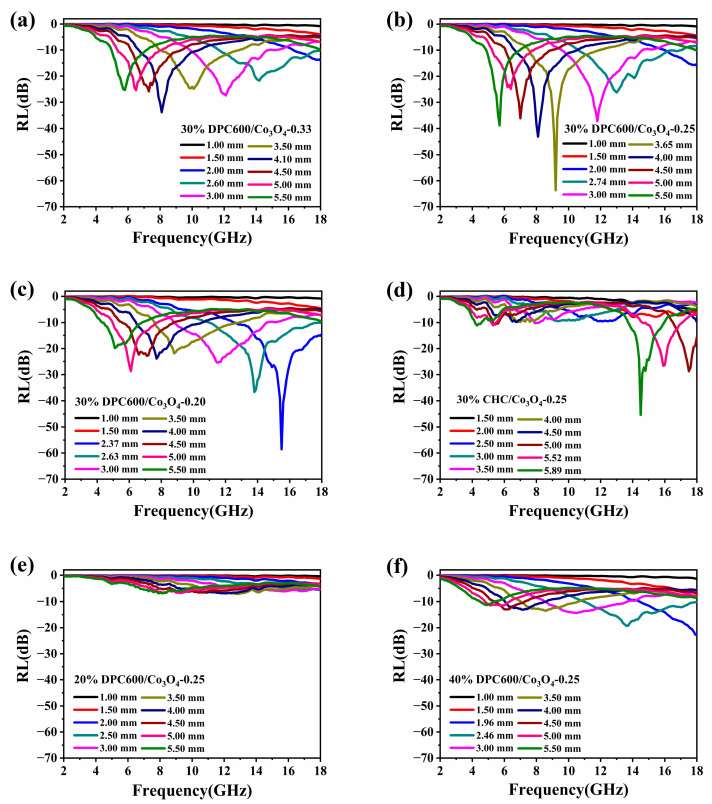
Characterization of the electromagnetic absorption properties of prepared samples: (**a**) 30% DPC600/Co_3_O_4_-0.33, (**b**) 30% DPC600/Co_3_O_4_-0.25, (**c**) 30% DPC600/Co_3_O_4_-0.20, (**d**) 30% CHC/Co_3_O_4_-0.25, (**e**) 20% DPC600/Co_3_O_4_-0.25, (**f**) 40% DPC600/Co_3_O_4_-0.25, the reflection loss values at different thicknesses.

The absorbing performance of each composite sample at different matched thicknesses is shown in Figure 4. The RL values of the samples with different thicknesses were simulated according to Equations (3) and (4), and for each absorber thickness, the maximum RL would occur at a certain frequency and would decrease as the absorber thickness increased. Due to the quarter-wavelength attenuation rule, the RL peaks of DPC600/Co_3_O_4_ hybrids shifted toward a lower frequency with an increase in thickness [40].

Figure 4 show the RL plots of the DPC600/Co_3_O_4_ hybrid with different Co_3_O_4_ nanoparticle loadings at thicknesses of 1.5–5.5 mm. It was confirmed by previous work and this work (Figure 4b,e,f) that the absorption performance is sensitive to the absorber filling ratio, with the best absorption effect at 30 wt.%. When the solid content was 30%, the maximum reflection loss value of DPC600/Co_3_O_4_-0.33 was −33.8 dB at a thickness of 4.10 mm (8.1 GHz), the maximum RL value of DPC600/Co_3_O_4_-0.25 was −63.7 dB at a thickness of 3.65 mm (9.2 GHz), and the maximum RL value of DPC600/Co_3_O_4_-0.20 was −58.6 dB at a thickness of 2.37 mm (15.5 GHz) (Figure 4a–c and Table 2). The higher the content of Co_3_O_4_, the lower the maximum absorption frequency. This result indicated the obvious effect of Co_3_O_4_ on reducing the absorption frequency.

In all samples, the sample of 30% DPC600/Co_3_O_4_-0.25 had the largest RL value, and it had the best wave absorption performance in the 5–12 GHz frequency band. Figure 4c displays the RL plot of DPC600/Co_3_O_4_-0.20; it can be seen that DPC600/Co_3_O_4_-0.20 showed an inferior EM wave absorption performance in the frequency range of 5–12 GHz, which may be associated with the high permittivity of DPC600/Co_3_O_4_-0.20.

Compared to DPC600, DPC600/Co_3_O_4_-0.25 had an improved maximum RL value and low frequency wave absorption performance [17]. The frequency corresponding to the maximum RL was reduced from 16.9 GHz to 9.2 GHz. Furthermore, DPC600/Co_3_O_4_-0.25 had a good wave absorption performance in a wide thickness range of 2.06 mm (2.78–4.84 mm), and the sample still maintained an RL value of less than −30 dB. However, the thickness change of DPC600 was only 0.33 mm (2.14–2.47mm). As a comparison, the commercial biomass carbon, CHC/Co_3_O_4_-0.25, only reached a maximum RL value of −45.4 dB at 14.5 GHz (Figure 4d), and the thickness changes maintained an RL value of less than −30 dB at only 0.07 mm. All these indicate that through the introduction of Co_3_O_4_, our porous DPC600 had greater practical application potential.

Impedance matching is the other key factor to be considered when designing absorbing materials [41,42]. To analyze the electromagnetic wave absorption performance of the DPC/Co_3_O_4_ nanocomposites, impedance matching was investigated. The impedance matching is given by Equation (5) as follows:

(5) $$Z=Z_{\mathrm{in}}/Z_{0}=\sqrt{\mu_{r}/\varepsilon_{r}}\tanh\left|j\left(2\pi fd/c\right)\sqrt{\mu_{r}\varepsilon_{r}}\right|\;$$

**Figure 5:**
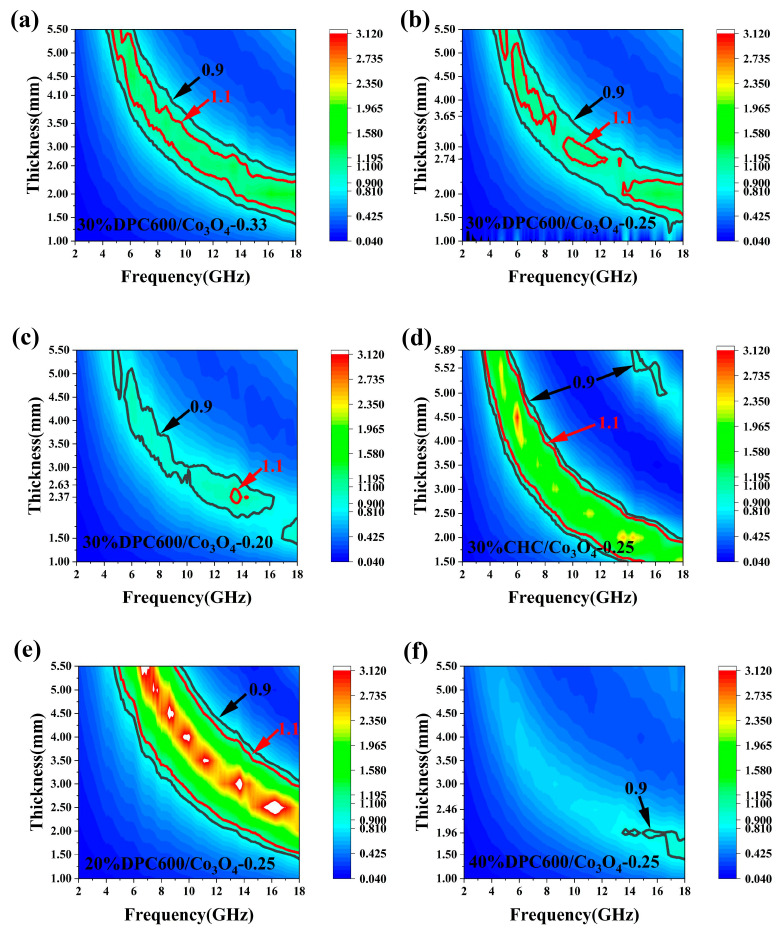
Three-dimensional representation of the impedance matching of prepared samples: (**a**) 30% DPC600/Co_3_O_4_-0.33, (**b**) 30% DPC600/Co_3_O_4_-0.25, (**c**) 30% DPC600/Co_3_O_4_-0.20, (**d**) 30% CHC/Co_3_O_4_-0.25, (**e**) 20% DPC600/Co_3_O_4_-0.25, (**f**) 40% DPC600/Co_3_O_4_-0.25.

As the Z value becomes closer to 1, fewer microwaves are reflected, and more microwaves are absorbed by the materials. The equation shows that impedance matching is related to Co_3_O_4_ loading, thickness, frequency, and solid content. From the equation, a lower relative complex permittivity (*ε_r_*) helps to obtain a suitable Z-value over a wide range of frequencies and thicknesses. The calculated Z-values of DPC/Co_3_O_4_ at different molar ratios and solid contents with frequency are shown in Figure 5. In addition, Z = 0.9 and Z = 1.1 are marked with black and red lines, respectively.

**Table 2:** Microwave absorption performance of each composite sample.

Sample	Loading Ratio (wt.%)	Maximum RL Value (dB)	Thickness (mm)	Frequency (GHz)	Frequency Range (GHz)	Effective Bandwidth (GHz)	Thickness Range with Maximum RL Less than −30 dB (mm)
DPC600	30	−60.0	2.30	16.9	10.6–17.9	7.3	2.14–2.47
DPC600/Co_3_O_4_-0.20	30	−58.6	2.37	15.5	11.0–17.9	6.9	2.15–2.81
DPC600/Co_3_O_4_-0.25	30	−63.7	3.65	9.2	10.6–17.0	6.4	2.78–4.84
DPC600/Co_3_O_4_-0.33	30	−33.8	4.10	8.1	11.7–18.0	6.3	4.04–4.21
CHC/Co_3_O_4_-0.25	30	−45.4	5.89	14.5	14.6–17.0	2.4	5.81–5.89
Co_3_O_4_/rGO [23]	50	−61.62	2.40	11.2	9.6–13.4	3.8	\
CoO_x_/CFs [24]	33	−45.16	1.50	13.4	\	\	\
Co_3_O_4_@C@PGC [25]	50	−32.3	2.30	11.4	10–13.4	3.4	\
Co_3_O_4_–C [43]	5	−51.8	2.00	15.8	13.2–17.8	4.6	\
Co@Co_3_O_4_/C [44]	30	−46.4	2.50	15.5	12.3–18	5.7	\

As seen in Figure 5a–c, the different molar ratios of Co_3_O_4_ and DPC600 seriously affected the magnitude of the Z-value of the composite; 30% DPC600/Co_3_O_4_-0.33 had a certain impedance matching effect in the frequency range of 5–18 GHz. As the Co_3_O_4_ loading decreased, 30% DPC600/Co_3_O_4_-0.25 and DPC600/Co_3_O_4_-0.20 had larger impedance matching areas, namely, from 0.9 to 1.1. However, at 5–12 GHz, the impedance matching of DPC600/Co_3_O_4_-0.20 was discontinuous, and Z = 1 was uncommon in this frequency range. This was primarily caused by the increased relative dielectric constant imaginary part ε″ due to the increase in carbon content ratio. As a result, while the sample had superior electromagnetic characteristics, its microwave absorption capability at 5–12 GHz fell short of 30% DPC600/Co_3_O_4_-0.25. The impedance matching of 30% DPC600/Co_3_O_4_-0.25 was substantially improved compared to the 30% DPC600 studied in previous work [17], which is attributed to the fact that the introduced Co_3_O_4_ magnetic particles increased the interfacial polarization of Co_3_O_4_ and DPC600 while also changing the electromagnetic parameters of the composite, making the *ε_r_* value closer to the *μ_r_* value.

Different paraffin ratios of the same absorbing material also affected the impedance matching performance. Figure 5b,e,f compare the impedance matching of DPC600/Co_3_O_4_-0.25 at different solid contents, and the impedance matching was poorer in the narrower range of Z values close to 1 at filling ratios of 20 wt.% and 40 wt.%. This was mainly because the different paraffin ratios changed the electromagnetic parameters and caused a great difference in the impedance matching performance. We also found that the performance of the 30% DPC600/Co_3_O_4_-0.25 was better than that of CHC/Co_3_O_4_-0.25 through impedance matching comparison (Figure 5b,d).

Finally, a comprehensive comparison revealed that 30% DPC600/Co_3_O_4_-0.25 had outstanding impedance matching performance in a larger frequency range, which was attributed to its suitable electromagnetic parameters.

## 4. Conclusions

D-fructose porous carbon materials were created through hydrothermal carbonization and KOH activation, and DPC600/Co_3_O_4_ composites were achieved by in situ growth of Co_3_O_4_ on it, and CHC/Co_3_O_4_ composites were created from CHC materials as a comparation. The introduction of Co_3_O_4_ magnetic particles increased the interface polarization and realized the magnetic loss through the resonance between materials and reduced the frequency of the maximum RL. The composite DPC600/Co_3_O_4_-0.25 had the best maximum RL value and low frequency wave absorption performance, with a molar ratio of Co_3_O_4_ to DPC600 of 1:3. The highest reflection loss RL value of −63.7 dB at 9.2 GHz was achieved for DPC600/Co_3_O_4_-0.25 with a filling ratio of 30% and a thickness of 3.65 mm, and the bandwidth was 6.4 GHz (10.6–17.0 GHz) when the thickness was 2.74 mm. Compared with samples without introduced Co_3_O_4_ (DPC600), this composite material had better wave absorption performance at a wide thicknesses range (2.06 mm compared to 0.33 mm, maintaining an RL value of less than −30 dB), the frequency of the maximum RL was reduced from 16.9 GHz to 9.2 GHz, which greatly broadens the frequency range in which the composite material can be used.

## Figures and Tables

**Table 1:** Pore structure and specific surface of DPC600 and CHC.

Sample	S_BET_ (m^2^g^−1^)	S_micropores_ (m^2^g^−1^)	S_external_ (m^2^g^−1^)	V_pore_ (cm^3^g^−1^)	A_size_ (nm)
DPC600	1824.3	1326.7	497.7	0.85	1.87
CHC	746.6	661.6	85.0	0.37	1.98

## Data Availability

Not applicable.
